# Exploration of the relationship between primary dysmenorrhea, pain perception, and menstruation-related quality of life in young women: a cross-sectional observational study

**DOI:** 10.3389/fgwh.2025.1521276

**Published:** 2025-02-11

**Authors:** Rebeca Del Prado, María García-Arrabé, Ángel González-de-la-Flor, Marta De La Plaza San Frutos, Jaime Almazán Polo, Fabien Guérineau, Cecilia Estrada-Barranco

**Affiliations:** Department of Physiotherapy, Faculty of Medicine, Health and Sports, Universidad Europea de Madrid, Madrid, Spain

**Keywords:** dysmenorrhea, pain, pain catastrophizing, quality of life, menstruation

## Abstract

**Background:**

This study explores the relationship between primary dysmenorrhea (PD), pain, pain catastrophizing, and menstruation-related quality of life in young women.

**Methods:**

A cross-sectional study was conducted involving 44 young women, both with and without PD. Various variables including pain intensity, pain catastrophizing, and menstruation-related quality of life were assessed using validated questionnaires. Correlation and regression analyses were performed to examine the relationships between the variables.

**Results:**

Significant associations were found between the presence of PD, tendency to catastrophize pain, and decreased menstruation-related quality of life. A high correlation was observed between pain intensity and catastrophizing, indicating mutual influence between these variables. Menstruation-related quality of life was affected in terms of health perception, psychological aspect, and symptoms among women with PD. The linear regression model demonstrated that catastrophizing explained 42.8% of the variance in menstruation-related quality of life.

**Conclusions:**

These findings underscore the importance of addressing dysmenorrhea in young women, as it significantly impacts their quality of life related to menstruation. Understanding the factors contributing to dysmenorrhea and its effects on quality of life can inform more effective, patient-centered treatment strategies.

## 1 Introduction

Primary dysmenorrhea (PD), also known as menstrual pain, is a common gynecological condition affecting young women during their reproductive years. It is characterized by pain in the lower abdomen accompanying menstruation and may be associated with other symptoms such as nausea, vomiting, fatigue, and lower back pain (1). The prevalence of PD among women of reproductive age is very high worldwide. Data shows percentages ranging from 64% in Mexico (2), 51% in China, 74.8% in Spain (3), 91.5% in Ireland (3), and 90.7% in Brazil (4). These high prevalence rates highlight the substantial burden of primary dysmenorrhea, which can adversely affect various aspects of women's lives, including work, education, and interpersonal relationships, ultimately contributing to a diminished quality of life (5).

The cause of PD is not clear. On one hand, increased prostaglandins at that point in the menstrual cycle may excite nociceptors and cause colicky pain due to increased uterine contractility (6). However, other authors suggest that menstrual pain is primarily associated with mechanisms unrelated to increased prostaglandins, such as inadequate uterine perfusion (7). In most cases, the use of Non-Steroidal Anti-Inflammatory Drugs (NSAIDs) can alleviate symptoms (8); however, 18% of women with PD do not respond positively to administration (9). Furthermore, the presence of PD has been associated with a higher risk of experiencing chronic pelvic pain (10), central sensitization syndrome (11), and even increased pain sensitivity with changes in the central nervous system (12).

On the other hand, the presence of symptoms and such sensitization may extend beyond the days of the menstrual cycle when menstruation occurs (11). Chronic menstrual pain may be associated with a higher risk of developing mood disorders such as depression and anxiety (13), as well as sleep problems (14). It may even result in distortion in attention and cognitive processing (15), tending to magnify or exaggerate pain perception.

Mood disorders are increasingly recognized as both a consequence and a contributing factor to chronic pain conditions. This bidirectional relationship between chronic pain and mood disorders not only exacerbates pain perception but also involves common neurophysiological mechanisms, such as dysfunction of the hypothalamic-pituitary-adrenal axis, alterations in monoaminergic neurotransmission, and increased systemic inflammatory mediators (16–18). Understanding these links is essential to, first, establishing a diagnostic approach, as well as identifying effective interventions that address both the pathophysiological and psychological aspects of primary dysmenorrhea, with the aim of interrupting the cycle of chronic pain and affective comorbidities.

In patients with chronic pain, in addition to the presence of mood disorders, an increase in catastrophizing has been observed as a maladaptive response to pain perception. Its impact has been studied in various types of chronic pain, showing a significant association with pain progression (19), quality of life (15), and intensity of the pain itself (20). In the case of women with PD, catastrophizing may exacerbate the problem and its consequences (21).

PD has a high prevalence and significant impact on the quality of life of affected women, and various studies have examined the influence of psychological factors, such as catastrophizing, in modulating the pain experience (15, 20, 22). According to the World Health Organization, quality of life is defined as “an individual's perception of their position in life in the context of the culture and value systems in which they live, and in relation to their goals, expectations, standards, and concerns” (23). The assessment of quality of life in this context requires the use of specific and validated tools that address the relevant domains for this population (24, 25).

The Specific Quality of Life Questionnaire Related to Menstruation (M-QOL-22) (26) is a validated and reliable instrument designed to measure quality of life associated with menstrual pain in Spanish-speaking populations. This questionnaire consists of three subscales that assess: (1) self-perception of the situation, (2) psychological aspects, and (3) symptom perception. The subscale structure allows for a more detailed and specific evaluation of the different factors that affect quality of life in women with PD.

Given that catastrophizing has been identified as a maladaptive psychological response that can amplify pain perception and impair quality of life, it is crucial to investigate how it specifically interacts with the domains assessed by the M-QOL-22.

Despite existing research on PD, few studies have specifically examined how catastrophizing influences menstruation-related quality of life in women affected by this condition. This gap in the literature represents a significant knowledge void that the present study aims to address. The primary objective of this study is to analyze the impact of PD on various aspects of quality of life, as well as on the presence of catastrophizing thoughts. Additionally, the study seeks to explore the relationship between physical factors, pain intensity, catastrophizing thoughts, and different domains of menstruation-related quality of life in order to gain a more comprehensive understanding of the phenomenon. Finally, a linear regression model will be established to identify the variables that influence the variance in menstruation-related quality of life.

## 2 Methodology

### 2.1 Study design

A cross-sectional observational study with a control group (CG) was conducted at the Universidad Europea de Madrid (UEM), Spain, between September 2022 and March 2023. The study received approval from the Research Ethics Committee of the European University, under the code CIPI/23.146, on April 20, 2023. To ensure methodological quality, the criteria proposed by the Strengthening the Reporting of Observational Studies in Epidemiology (STROBE) guidelines (27) were followed.

### 2.2 Participants

Participants were recruited from students and staff at the European University of Madrid.

Recruitment of participants was done through posters placed in public spaces and messages shared on social media. These materials provided detailed information about the study, encouraged participation, described the inclusion criteria, and clarified that participation required attendance at the lab for only one day. This approach ensured transparency, clarity and broad accessibility, while maximising visibility.

Inclusion criteria for the dysmenorrhea group required participants to be women over 18 years old, within reproductive age, nulliparous, and diagnosed with PD by a physician from the UEM medical service. Exclusion criteria included having a previously diagnosed osteoarticular disorder, using hormonal contraceptives or intrauterine devices (IUDs), or having a diagnosis of endometriosis or any other urogynaecological condition, any systemic pathology or chronic pain, and any surgery in the last 6 months. CG participants had to fulfill the same criteria as the dysmenorrhea group, except they were required to be free of PD.

Participants were subsequently classified into two groups based on the physician's diagnosis: 22 women diagnosed with PD and 22 women without PD.

### 2.3 Methods

Upon acceptance of participation, participants completed a questionnaire including questions about the regularity of their menstrual cycle and the date of their last period. Additionally, participants completed the Pain Catastrophizing Scale (PCS) and the M-QOL-22. Data on daily lifestyle habits such as smoking habit, age, height, weight, and body mass index (BMI) were also collected. Pain intensity during the first day of the cycle was quantified using the visual analogue scale (VAS) (28). Participants completed the questionnaires under the supervision of a physiotherapist, who was available to address any questions that arose during the process.

To obtain information about quality of life and menstruation-related catastrophizing, several validated instruments were used:

To assess catastrophizing associated with menstrual pain, the PCS (29) was used. It consists of 13 items expressing different thoughts and emotions that may be associated with pain. The items are divided into three spheres: magnification, rumination, and helplessness. Each item is rated on a 5-point Likert scale, from (0) not at all to (4) all the time. Higher scores indicate a higher degree of pain catastrophizing (30).

The M-QOL-22 (26) was specifically developed to assess the impact of menstrual pain on the quality of life of women of childbearing age. It addresses perceptual, psychological, and symptomatic aspects related to menstruation. It consists of 22 items, each rated on 3-point Likert scales, where higher scores indicate poorer quality of life. It is further divided into three subscales exploring three aspects of quality of life: perception of health and physical and functional well-being, psychological and cognitive well-being, and symptomatology associated with menstruation (26). This questionnaire has demonstrated excellent test-retest reliability [intraclass correlation coefficient (ICC) = 0.9] and excellent internal consistency (Cronbach's Alpha = 0.917).

### 2.4 Sample size calculation

The sample size estimation was calculated using a commonly accepted rule-of-thumb method for regression analyses, which suggests having a minimum of 10 data points per predictor variable to ensure adequate power for detecting associations and conducting factor analysis (31, 32). Following this recommendation, a minimum requirement of at least 40 participants per model was determined (33).

### 2.5 Statistical analysis

All statistical analyses were performed using SPSS software v.29 for Windows (IBM SPSS, Armonk, NY, USA). Initially, the normality of data distribution was assessed using the Shapiro–Wilk test and histograms, while variance homogeneity was examined using the Levene test. *P*-values less than.05 were considered indicative of non-normal distribution, while *P*-values greater than.05 suggested normal distribution. Descriptive analysis was then conducted to characterize the sample, with measures of central tendency and dispersion presented as mean and standard deviation for normally distributed variables, and median and interquartile range for non-normally distributed variables, respectively.

Differences between cases and controls were evaluated using the Student *t*-test for independent samples for quantitative data, with a significance threshold set at *p* < .05.

Associations between variables were estimated by creating a matrix with Pearson correlation coefficients (r). Correlation levels were established as follows: Non-existent (*r* < 0.1); Low (*r* = ≥0.1 < 0.3); Moderate (*r* = ≥0.3 < 0.5); High (*r* = ≥0.5 < 0.7), Very high (*r* = ≥0.7 < 0.9), and Almost perfect (*r* ≥ 0.9) (32). Additionally, r coefficients were used to detect multicollinearity and shared variance (defined as *r* > 0.80) to mitigate the risk of bias and overestimation in the regression model.

A multivariate linear stepwise regression model was developed to assess menstruation-related quality of life. Variables demonstrating the strongest correlation, without shared variance and achieving statistical significance (*p* < .05), were sequentially included in the stepwise regression model. The significance criterion for the critical F value was set at *p* < .05, with changes in adjusted variance (adjusted R2) reported at each step to assess the individual variance contribution (26, 30, 31).

## 3 Results

A total of 44 women were recruited randomly from those who volunteered to participate in the study. After being diagnosed by the university medical service, they were divided into 22 participants in the PD group and 22 participants in the CG. Table 1 summarizes the anthropometric characteristics of the study participants and the intensity of pain on the first day of menstruation, collected using the VAS. The data are distributed across the total sample group and the PD and control subgroups.

**Table 1:** Anthropometric and pain related characteristics of the study participants.

Variables	Total sample (*n* = 44)	CG (*n* = 22)	DG (*n* = 22)	*P*-value	MD (95% CI)
Age	26.16 ± 6.97	25.14 ± 7.41	27.18 ± 6.51	0.337	2.05 (−2.04; 2.10)
Weight	61.24 ± 9.88	61.16 ± 10.89	61.32 ± 9.02	0.958	−0.16 (−6.24; 5.92)
Height	164.91 ± 6.46	163.82 ± 6.86	166.00 ± 5.98	0.267	−2.18 (−6.10; 1.74)
BMI	22.44 ± 2.97	22.70 ± 3.31	22.18 ± 2.64	0.567	0.52 (−1.30; 2.34)
VAS (0–10)	5.68 ± 2.81	3.23 ± 1.38	8.14 ± 1.28	**<0**.**001**	−4.91 (−5.72; −4.10)

CG, control group; DG, dysmenorrhea group; MD, mean difference; CI, confidence Interval; BMI, body mass index; VAS, visual analogue scale. Bold values indicate *P* < 0.05.

Regarding age, weight, height, and body mass index (BMI), no significant differences were found between the CG and the PD group (*p* > 0.05 for all comparisons). However, significant differences were found between the groups regarding pain intensity. The PD group exhibited considerably higher pain intensity (8.14 ± 1.28) compared to the CG (3.23 ± 1.38) (*p* < 0.001). This difference in pain intensity between the groups was statistically significant (mean difference: −4.91 points; 95% CI: −5.72; −4.10), highlighting the significant influence of PD on pain experience in the study participants.

Table 2 presents data on pain catastrophizing and menstruation-related quality of life (M-QOL-22) in the total sample, as well as in the control and PD subgroups. Significant differences between the groups were found in terms of pain catastrophizing. The PD group exhibited a higher score on the PCS, with an average of 25.55 (±9.60), compared to the CG with an average of 7.41 (±8.51) (*P* < 0.001). This difference in PCS between the groups was statistically significant, with a mean difference of 18.14 points (95% CI: −23.65; −12.62), indicating a greater tendency to catastrophize pain in women with PD. Statistically significant differences were also found between the two groups in all three subscales: Rumination with a mean difference of −6.63 points (95% CI: −8.60; −4.67), Magnification, −2 points (95% CI: −3.48; −0.52), and Helplessness −9.5 points (95% CI: −12; −6.99).

**Table 2:** Pain catastrophizing and menstruation-related quality of life data of the total sample, control and dysmenorrhea group.

Variables	Total sample (*n* = 44)	CG (*n* = 22)	DG (*n* = 22)	*P*-value	MD (95% CI)
PCS (0–52)	16.48 ± 12.83	7.41 ± 8.51	25.55 ± 9.60	**<0**.**001**	−18.14 (−23.65; −12.62)
PCS (Rumination)	6.04 ± 4.64	2.62 ± 3.18	9.36 ± 3.29	**<0**.**001**	−6.63 (−8.60; −4.67)
PCS (Magnification)	3.04 ± 2.61	2.05 ± 2.42	4.05 ± 2.46	**0**.**09**	−2 (−3.48; −0.52)
PCS (Helpnessless)	7.38 ± 6.3	2.64 ± 3.32	12.14 ± 4.79	**<0**.**001**	−9.5 (−12; −6.99)
M-QOL-22 (0–66)	25.16 ± 17.20	15.55 ± 13.70	35.91 ± 13.57	**<0**.**001**	20.36 (−28.66; −12.07)
M-QOL (Perception)	11.75 ± 8.11	7.64 ± 7.21	15.86 ± 6.87	**<0**.**001**	8.23 (−12.51; −3.94)
M-QOL Pyschology)	9.07 ± 6.17	5.41 ± 4.91	12.73 ± 5.07	**<0**.**001**	7.32 (−10.35; −4.28)
M-QOL (Symptoms)	4.91 ± 3.67	2.50 ± 2.18	7.32 ± 3.26	**<0**.**001**	4.82 (−6.50; −3.13)

CG, control group; DG, dysmenorrhea group; MD, mean difference; CI, confidence Interval; PCS, pain catastrophizing scale; M-QOL, menstruation related quality of life. Significance was set at *P*-value <0.05 (in bold).

Regarding menstruation-related quality of life (M-QOL), significant differences were observed between the groups in the perception, psychology, and symptoms subscales. The DG reported significantly lower scores in all M-QOL subscales compared to the CG (*P* < 0.001 for all comparisons). Specifically, the DG showed lower average scores in M-QOL (Perception), M-QOL (Psychology), and M-QOL (Symptoms) compared to the CG, with mean differences of 8.23 points (95% CI: −12.51; −3.94), 7.32 points (95% CI: −10.35; −4.28), and 4.82 points (95% CI: −6.50; −3.13) respectively.

The correlation data presented in Table 3 show significant relationships between the evaluated variables in the sample of young women in the DG, while in the CG no significant correlations were found in any of the studied variables. In the DG, a very high significant correlation was observed between pain intensity and pain catastrophizing (PCS) [*r* = 0.73 (*p* < 0.001)], high with the PCS (Rumination) subscale [*r* = 0.57 (*p* = 0.005)] and PCS (Magnification) [*r* = 0.662 (*p* < 0.001)], and very high with the PCS (Helplessness) subscale [*r* = 0.73 (*p* < 0.001)]. Moderate correlations were found between pain intensity and quality of life [*r* = 0.45 (*p* = 0.03)], as well as the different subscales: M-QOL (Perception) [*R* = 0.438 (*p* = 0.04)], M-QOL (Psychology) [*r* = 0.42 (*p* = 0.05)], and M-QOL (Symptoms) [*r* = 0.46 (*p* = 0.03)]. Also, with the different subscales of catastrophizing (*r* = 0.784, *P* < 0.01), as well as between pain catastrophizing and the subscales of menstruation-related quality of life (M-QOL) (*r* = 0.448–0.689, *P* < 0.01). A high level of correlation was also found between quality of life and catastrophizing [*r* = 0.65 (*p* = 0.001)]. The correlation between the total M-QOL-22 score and the different subscales of the PCS was significant in all cases and moderate for rumination [*r* = 0.47 (*p* = 0.027)] and high for magnification [*r* = 0.69 (*p* < 0.001)] and helplessness [*r* = 0.62 (*p* = 0.002)]. The other subscales of quality of life and catastrophizing also correlated positively with each other, except M-QOL (Psychology) and M-QOL (Symptoms) with PCS (Rumination) whose correlations were not significant.

**Table 3:** Correlation analysis of evaluated variables in the dysmenorrhea group.

	VAS	PCS	PCS (Rumiation)	PCS (Magnification)	PCS (Helpnessless)
VAS	**-**	0.730**	0.570**	0.662**	0.730**
M-QOL-22	0.450*	0.650**	0.470*	0.690**	0.620**
M-QOL (Perception)	0.438*	0.660**	0.530**	0.670**	0.630*
M-QOL (Psychology)	0.420*	0.560**	n.s.	0.640**	0.550**
M-QOL (Symptoms)	0.460*	0.497**	n.s.	0.480**	0.553**

PCS, pain catastrophizing scale; M-QOL, menstruation related quality of life. BMI, body mass index; VAS, visual analogue scale. n.s., non-significant.

* *p* < 0.05.

** *p* < 0.01.

Given the high correlation between catastrophizing and quality of life, a linear regression model was conducted to further explore this relationship. Table 4 shows the parameters of the linear regression models. A model was established in which PCS scores could substantially explain (42.8% of the variance) the score on the M-QOL-22 scale for the total sample.

**Table 4:** Summary of the regression analyses to determine predictors of M-QOL-22.

	Predictor Outcome	Adj *R*^2^	B	SE B	95% CI	*t*	*P*
M-QOL-22	PCS	0.428	0.879	0.665	0.5713; 1.187	5.765	<0.001

B, standarized beta coefficient; SE B, standard error of beta.

## 4 Discussion

These findings indicate a significant relationship between PD, pain, pain catastrophizing, and decreased menstrual-related quality of life in young women. The presence of PD was associated with a greater tendency to catastrophize pain, both in the aspect of rumination, magnification and helplessness, and a worse menstrual-related quality of life in terms of perception, psychology and symptoms with respect to CG. These data reveal a worrisome reality given the enormous prevalence of PD in women of childbearing age worldwide.

Our findings align with previous research demonstrating a connection between heightened pain perception and increased catastrophizing, which in turn diminishes quality of life (21). One possible explanation for this relationship is that intensified pain perception amplifies the psychological impact of PD, leading to maladaptive coping strategies like catastrophizing, creating a feedback loop that worsens both pain intensity and emotional distress (20, 21). Central sensitization has also been suggested as a mechanism driving this cycle, as it has been implicated in both chronic pelvic pain and pain catastrophizing (11). These interrelated mechanisms underscore the need for multidimensional treatment approaches. Interventions targeting catastrophizing behaviors could disrupt this feedback loop, reducing pain perception and improving quality of life. Further studies should examine whether such interventions can mitigate the effects of central sensitization in PD.

Menstrual pain intensity may be mediated by physiological factors such as the presence of heavy bleeding, certain dietary habits (34), or vitamin D levels (35). However, our findings corroborate that PD is a multifactorial phenomenon in which psychological, social, and cultural factors coexist that can influence the perception of such pain, such as educational level, adequate information, or economic status (35). Psychosocial factors, including parental education and care regarding girls’ menstrual health (36), may also play a role in shaping the perception of pain (21). Moreover, although none of the participants were menstruating when completing the questionnaires, our results align with those of Gagnon et al. (11), suggesting that quality of life alterations and increased catastrophizing persist throughout the menstrual cycle.

No relationships between catastrophizing and quality of life were found in any of the subscales in the CG, unlike the DG. This may be explained because, while both variables may be influenced by other factors in CG women, the level of quality of life, in the absence of pain, is high enough for the correlation not to be significant.

In this study, the PCS was used, allowing the analysis of different aspects of catastrophization such as rumination, magnification, and helplessness. Rumination has been more related to the cognitive and attentional aspect, focusing on the phenomenon, magnification is related to an evaluation of the event as a threat, and helplessness is related to a sense of defenselessness or incapacity to cope, following a secondary evaluation (37, 38). According to our results, all three subscales showed high significant correlations with pain intensity. Although some studies, such as that by Cosic et al. (39), reported higher levels of catastrophization during the first day of the menstrual cycle, the participants in this study were not in this moment and yet reported higher levels of catastrophism.

The scale used in this study, the M-QOL-22, is a scale developed and validated in Spanish that specifically studies quality of life in relation to menstruation. To our knowledge, the relationship between quality of life restricted to aspects related to menstruation and catastrophization had not been previously studied. The M-QOL-22 subdivides quality of life spheres into three different aspects: health perception, psychological, and symptom-related. Our findings show that all three spheres are affected by pain intensity in the case of women with PD. However, rumination only showed significant correlations with the health perception subscale, not with the psychological or symptomatology subscale. This could be explained by the fact that focusing attention on one's own health status modifies the perception we have of it. These results coincide with those found by Craner et al. (40), where rumination was the only one of the three subscales that was not clearly associated with the other variables studied.

None of the anthropometric variables showed significant correlations with quality of life or catastrophization. However, the relationship between total scores of PCS and M-QOL-22 in the total sample and in the PD group is established by the regression model obtained in this study. These data allow us to delve into the capacity of the PCS to predict values of quality of life, which may allow for establishing strategies for catastrophization that improve the quality of life of women with PD.

These findings underscore the need for a comprehensive approach to managing dysmenorrhea (PD), recognizing its significant impact on menstrual-related quality of life and pain perception. Effective management should integrate physical and psychological interventions to address the multifaceted nature of PD. Interventions targeting pain catastrophizing, alongside symptom alleviation strategies, hold promise for reducing pain intensity and improving overall well-being in affected women. Chronic pain management strategies should not only aim to reduce symptoms but also foster adaptation and coping, emphasizing a holistic approach to improving quality of life (41).

Recent evidence supports the efficacy of manual therapy and pelvic floor exercises in reducing pain associated with PD (42), while cognitive-behavioral approaches targeting catastrophizing may further enhance outcomes. Future studies should explore the integration of these strategies into a holistic treatment model to evaluate their combined impact on pain management and quality of life. Additionally, pelvic manipulation has demonstrated potential in altering pain perception in PD (43), highlighting the value of integrating manual therapy into routine care. Future research should focus on evaluating the effectiveness of such multidisciplinary interventions in clinical settings.

Expanding research to explore the benefits of physiotherapeutic techniques, cognitive-behavioral therapies, and lifestyle modifications—including dietary changes and physical activity—would provide valuable insights into PD management. Furthermore, assessing these interventions across different phases of the menstrual cycle could deepen our understanding of their efficacy and inform more personalized treatment strategies.

This study is not without limitations, which should be carefully considered when interpreting the findings. First, the cross-sectional design of the study allows for the identification of associations but precludes establishing causal relationships between variables. Second, while validated questionnaires were used to mitigate concerns about the accuracy of self-reported measures for assessing musculoskeletal conditions, the inherent limitations of self-reports, such as potential recall or response bias, remain. Third, the relatively small sample size (*n* = 44) limits the statistical power of the study and may reduce the generalizability of the findings. Future research should include larger, more diverse populations to enhance external validity and provide a more comprehensive understanding of these relationships. Finally, while all participants were in a similar phase of the menstrual cycle, which strengthens group comparisons, the study did not capture longitudinal data across different phases of the cycle. It would be valuable to replicate the study with a longitudinal design to explore the evolution of variables throughout the menstrual cycle.

## 5 Conclusion

Women with PD present lower quality of life related to menstruation both globally and in the perception of health status, psychological aspect and symptoms associated with menstruation, as well as higher levels of catastrophizing, the variables being related to each other. The linear regression model showed that catastrophizing explained 42.8% of the variance in menstruation-related quality of life.

## Data Availability

The raw data supporting the conclusions of this article will be made available by the authors, without undue reservation.
